# A System for Generating Customized Pleasant Pedestrian Routes Based on OpenStreetMap Data

**DOI:** 10.3390/s18113794

**Published:** 2018-11-06

**Authors:** Tessio Novack, Zhiyong Wang, Alexander Zipf

**Affiliations:** GIScience Research Group, Institute of Geography, Heidelberg University, 69120 Heidelberg, Germany; zhiyong.wang@uni-heidelberg.de (Z.W.); zipf@uni-heidelberg.de (A.Z.)

**Keywords:** volunteered geographic information, pedestrian routing, human–computer interaction

## Abstract

In this work, we present a system that generates customized pedestrian routes entirely based on data from OpenStreetMap (OSM). The system enables users to define to what extent they would like the route to have green areas (e.g., parks, squares, trees), social places (e.g., cafes, restaurants, shops) and quieter streets (i.e., with less road traffic). We present how the greenness, sociability, and quietness factors are defined and extracted from OSM as well as how they are integrated into a routing cost function. We intrinsically evaluate customized routes from one-thousand trips, i.e., origin–destination pairs, and observe that these are, in general, as we intended—slightly longer but significantly more social, greener, and quieter than the respective shortest routes. Based on a survey taken by 156 individuals, we also evaluate the system’s usefulness, usability, controlability, and transparency. The majority of the survey participants agree that the system is useful and easy to use and that it gives them the feeling of being in control regarding the extraction of routes in accordance with their greenness, sociability, and quietness preferences. The survey also provides valuable insights into users requirements and wishes regarding a tool for interactively generating customized pedestrian routes.

## 1. Introduction

Conventional routing services are able to find the shortest or fastest route from the origin to the destination. Pedestrians, however, frequently consider other aspects besides the shortest distance when walking for transport or as part of their recreational activities [1,2,3,4,5]. The related literature consistently agrees that pedestrian route choice is mainly influenced by six factors, namely, (1) distance to the destination; (2) feeling of safety; (3) intelligibility of the route; (4) general aesthetics of the built-up environment; (5) accessibility to locations of interest; and (6) the presence of green areas and the avoidance of air and noise pollution [6,7,8].

In recent years, a few pedestrian routing systems that consider these factors have been proposed. However, they rarely take into account the fact that the influence of some of these factors in pedestrian route choices is highly dependent on the time of day. For example, for the sake of safety, users might want to avoid green areas and streets with few or no open venues during the night. Moreover, pedestrians are individuals with different physical conditions and preferences regarding aspects of the environment. The purpose of the trip as well as the individual’s mood and company are circumstantial aspects that also strongly influence his choice of a walking route [9,10,11,12]. This leads us to believe that no pedestrian route will equally satisfy different individuals in different situations to the same extent.

As the additional distance (in relation to the shortest route) that pedestrians are willing to walk for the sake of safety, environmental pleasantness, etc. varies a lot according to their preferences and circumstances, pedestrian routing systems should enable users to request customized, situation-specific routes. Furthermore, they should inform the relevant route parameters for users to assess and compare the shortest and customized routes. This would assist them in their decision on whether to take one of the two routes or to further edit the preference settings in order to extract a more satisfying customized route. The availability of such a tool might motivate people to more often choose walking over other modes of transportation, which is known to be beneficial to the individual’s physical and mental health [13,14].

In this paper, we present and evaluate the concept of a pedestrian routing system that enables users to consider factors and quantify their influences on the extraction of walking routes. At present, these factors are limited to the street length, greenness, sociability, and quietness. Since our aim is to make this application operational worldwide by integrating it into OpenRouteService (https://maps.openrouteservice.org), a widely used open-source navigation-focused GIS web-service, we quantify these factors in simple, but effective processing ways based entirely on data from OpenStreetMap (OSM).

The remainder of this paper is organized as follows. Section 2 gives an overview of related works. In Section 3, the overall concept of the system is introduced. The quantification of the streets’ greenness, sociability, and quietness as well as the system’s routing cost function are also described in Section 3. Section 4 presents the user-survey designed to investigate the users’ perceptions on the system. Section 5 describes the experiments performed, and Section 6 presents and discusses the obtained results. Lastly, an outlook discussion is provided in Section 7.

## 2. Related Work

In a context where the wide range of web-map services that are becoming part of our lives is fastly growing, routing applications that consider the different factors that influence pedestrian route choice have been proposed and developed.

Focusing on the feeling of safety in pedestrian route choice, Bao et al. [15,16] proposed a pedestrian routing algorithm that considers the street lighting condition and the sidewalk width. Each street receives a score based on these two variables and their associated weights. The weights are different for day and night-time and were defined based on a survey with 25 participants. The authors also proposed a simple heuristic for reducing the total number of turns in the proposed route, thus increasing its comprehensiveness. With the same aim of generating safe routes during the night-time, Miura et al. [17] focused on the illumination aspect of streets as well. They developed a system that proposes pedestrian routes considering their length as well as the light intensity of streets sensed by a network of wireless sensor devices. They evaluated their system intrinsically with computer simulations. By combining OSM and authoritative geo-data, Keler and Mazimpaka [18] proposed a safety index to map dangerous areas and an approach for weighting road segments according to that map. The weighted road network was then used to compute safe routes during the night-time.

Pedestrians frequently choose their routes considering the ease of way-finding. As landmark-assisted way-finding has been shown to be efficient, in recent years, researchers have endeavoured to identify what makes landmarks identified as such, how to extract them in geo-datasets, and how to integrate them into pedestrian navigation systems. Schroder et al. [19] conducted an empirical study to identify the criteria to measure the salience of features in urban environments with the intention of using them as directional aids within route descriptions. Besides visibility and direction of approach, name, size, age, and colour were identified as important landmark features. Furukawa and Nakamura [20] also proposed a route planning algorithm that considers the visibility and “recognizability” of landmarks as a way to improve route understanding and thus reduce pedestrian anxiety. Roussel and Zipf [21] also proposed a pedestrian navigation service that assists way-finding based on landmarks. They evaluated the availability of landmarks in the OSM database and the suitability of these landmarks for pedestrian way-finding.

The overall pleasantness of a route is also a factor that influences pedestrian route choice [7]. Peregrino et al. [22] suggested the application of sentiment analysis techniques on Twitter data as a way to infer people’s opinions about Foursquare venues (e.g., pubs, museums, monuments). The authors then argued that the venues and their associated opinions could support finding routes that provide users with positive social experiences regarding their venue type interests. Kim et al. [23] also performed a sentiment analysis on geo-tagged tweets with the aim of extracting friendly and enjoyable pedestrian routes. The authors found a strong correlation between the tweet frequency and positiveness towards an area as estimated from official crime rate data. They created routes using the Google Maps API and then moved and added way-points so that the routes deviated from areas of high negative sentiment. Quercia et al. [24] aimed to automatically generate routes that were not only short, but emotionally pleasant as well. They relied on a crowd-sourcing platform (urbangems.org) for the evaluation of photos from streets with respect to their beauty, happiness, and quietness. After aggregating these evaluations to 200 × 200 m grid cells, they extracted the route with the best trade-off between distance and pleasantness through an optimization procedure that evaluated the average pleasantness of the *k*-shortest routes from origin to destination. Gavalas et al. [25] proposed a route planning app that enables tourists to combine scenic walking routes with subjective points-of-interest. Although the app is able to recommend routes that arguably maximize the users’ subjective satisfaction, the application is based on geo-data collected specifically for Athens (Greece). Unfortunately, the app was developed for a specific smartphone operational system, and it is not free of charge.

Environmental factors are also important in pedestrian route choice, as pedestrians frequently seek to reduce their exposure to different urban stressors. Moelter and Lindley [26] generated 100,000 home to primary school trips and computed the shortest route for each trip as well as the optimized route with respect to the least exposure to air pollution. Their results suggest that a decrease in exposure compensates for an increase in the route’s length, thus motivating the development of a route planning tool dedicated to the minimization of air pollution exposure. With the aim of reducing pedestrians’ heat stress, Russig and Bruns [27] proposed a route planner system which is able to find a route with minimal heat exposure.

To the best of our knowledge, our proposed system is the first to enable users to interactively and intuitively set the importance of different factors in the computation of pedestrian routes. As mentioned, the system is entirely based on OSM data, which makes it operational in any part of the world where OSM data is sufficiently available and reliable. Being aware of the advantages of using OSM as the dataset for routing applications, different researchers have focused on the analysis of the fitness-for-purpose of OSM data for that aim. Zielstra and Hochmair [28] investigated different proprietary and collaborative geo-datasets for cities in Germany and the US regarding the length of the shortest pedestrian routes. They concluded that OpenStreetMap was, already six years ago, relatively complete and thus usable for effective pedestrian routing. Recently, Mobasheri [29] investigated the fitness-for-purpose of OSM data for the routing of wheelchair users and individuals with reduced mobility. Based on their analysis, they concluded that the required information about sidewalk characteristics is frequently missing. Roussel and Zipf [21] affirmed that the effectiveness of extracting landmarks from OSM for pedestrian routing is relative and varies a lot from place to place. They, however, stressed that landmarks can be used in navigation instructions in urban areas with sufficient OSM data. The authors also stressed that OSM data completeness tends to increase with time.

## 3. General Concept of the System

**Figure 1:**
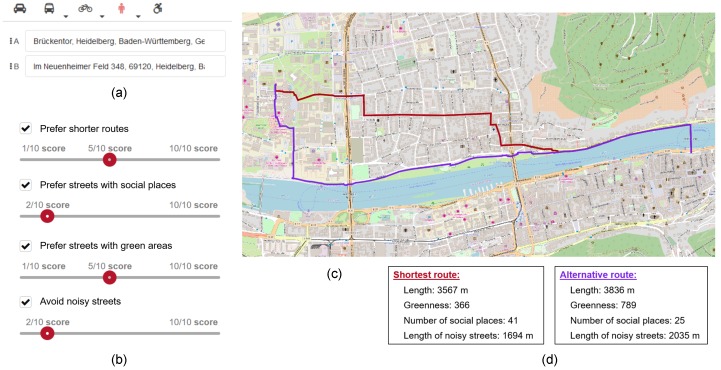
Main components of the proposed pedestrian routing system. (**a**) Origin and destination addresses are defined by the user. (**b**) Slide-bars to set the strength of influence of the different factors in the extraction of the customized route. (**c**) The system’s screen displays the shortest route (in red) and the customized route (in purple) over the OpenStreetMap layer. (**d**) A quantitative comparison between the shortest and customized routes in regard to the four factors is provided. The greenness variable is the street segment’s length multiplied by the relative area of the green areas inside its viewshed (see Section 3.2).

Typically, routing systems are based on a graph whose nodes represent street junctions and edges represent street segments. The edges may or may not be directed depending on the mode of transportation and the street’s traffic direction(s). The graph’s edges are associated with weights representing the street segments’ lengths and speed limits. In our proposed system, the weight of each edge *i* is a function of different variables *x*, each of which represents one of the four factors that might influence a pedestrian’s route choice. As mentioned, presently, these factors are the streets length, greenness, sociability, and quietness. Each variable *x* is normalized (notated as $\hat{x}$) by dividing each of its values by the maximum value observed at our test-site (see Section 5). Then, $\hat{x}$ is multiplied by a user-defined weight *w_x_* ∈ [0,10]. Finally, the four terms, $\hat{x}$*_i_w_x_*, are summed into a final overall weight for each street segment *i*. As in other routing systems, an algorithm minimizes the summation of the final weights in the graph’s path from the origin to the destination. What is special about this system is that the user is able to define the weights *w_x_* of each factor *x* in a interactive way by moving a slide-bar discretized into 11 levels from 0 to 10. The slide-bar configuration represents the subjective preferences of the user regarding the four factors that the system considers. Users may alter the slide-bar’s configuration for each trip according to their mood and other circumstantial aspects, such as weather conditions, time of day, purpose of the trip, etc. Figure 1 depicts the main interface components of the system. Besides visualizing the shortest and customized routes on the screen, users are able to see a quantitative comparison between the two routes in regard to the four factors. That means they are informed of the extra distance of the customized route (in relation to the shortest route) as well as how much more pleasant it is with respect to the other three factors.

### 3.1. Why These Factors?

The interactive routing system presented above is able to extract customized pedestrian routes considering the street length, greenness, sociability, and quietness. Below, we briefly discuss why the last three of these four factors were chosen.

Besides promoting outdoor activities like walking, cycling, and doing sports [30,31], green areas are known to enhance the feeling of calmness and tranquility, thus having a positive effect on people’s psychological health [32]. Furthermore, green areas also mitigate the negative effects of heat discomfort as well as air and noise pollution [33,34].

Places of social interaction are also associated with physical and mental health benefits among urban dwellers [35,36]. The possibility of social interaction is known to be correlated with the number, variety, and attractiveness of leisure and service destinations [37,38]. Although the influence of such destinations on pedestrians route choice might be stronger on individuals in recreational situations, Sugiyama et al. [39] and Cerin et al. [40] also found consistent associations between the route preferences of pedestrians in utilitarian situations and the presence and proximity of retail and service destinations.

Urban dwellers, and pedestrians specifically, are exposed to noise and air pollution, which negatively affect people’s well-being and may cause different health problems [41,42,43]. Air and noise pollution are strongly correlated because, in urban areas, both are mainly produced by road traffic [34,44]. Therefore, our pedestrian routing system gives users the option of preferring roads with less traffic. As mentioned earlier, the extent to which such roads should be avoided is interactively defined by users through a slide-bar.

It is important to emphasize that these factors are frequently interdependent. For example, streets with more road traffic often have more service and leisure destinations. Also, green areas like parks and squares become, at certain times of the day, places of social interaction. The feeling of safety and the aesthetics of built-up structures, two of the factors that also influence pedestrian route choice, are frequently associated with streets with an abundance and variety of services and leisure destinations. The main reason for this is the personalized parts of the streetfronts undertaken by business owners, the feeling of territorial control they transmit, and the permeability of the facades through shop windows are aspects that contribute to the feeling of safety and general perception of cleanness and good maintenance of building facades [37]. Furthermore, parts of the street personalized by business owners are negatively correlated with the presence of litter, vandalism, and graffiti [45].

### 3.2. How the Factors Are Extracted and Integrated

In this section, we explain how the greenness, social, and quietness factors were defined and quantified for each street segment based only on OSM data. We also explain how these factors were then integrated into a routing cost function.

**Figure 2:**
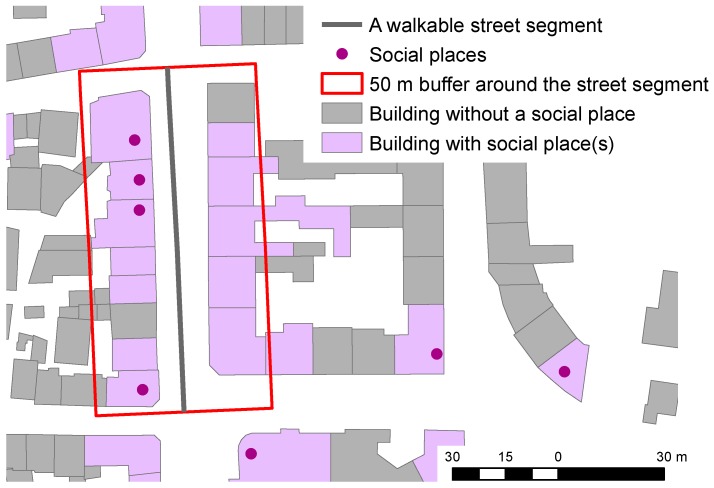
Example of a street segment and a 50 meter buffer zone around it. OpenStreetMap features intersecting the buffer and containing at least one of the tags indicate third places (Table 1) and were considered for the measurement of the street segment’s sociability.

Although the sociability of streets is difficult to measure and is, to a large extent, subjective for each person, streets that promote meetings and interactions with friends, acquaintances, and strangers are known to strongly correlate with the presence of so-called *third places*. Third places are places other than the individual’s home or working place [46]. Different studies have described the sociability of streets and neighbourhoods based on the presence of third places [37,38]. In this work, third places were extracted by selecting OSM nodes, ways, and relations containing at least one of the tags (i.e., key = value pairs) presented in Table 1. The sociability of street segments was computed by firstly defining a 50 m buffer around each street segment and then counting the number of OSM features intersecting this buffer with at least one of the tags listed in Table 1. This process is graphically depicted in Figure 2. Finally, the sociability factor was computed for each street segment by dividing its length by the number of third place features intersecting the buffer. Note that the list of OSM tags from Table 1 can easily be extended and edited without changing the other steps of the algorithm, which can also be applied to describe the cultural, historical, and architectural value of street segments. Aside from considering OSM tags related to those kinds of places instead of to social ones, this factor can be computed with the same processing chain as the one applied to describe the streets’ sociability.

**Figure 3:**
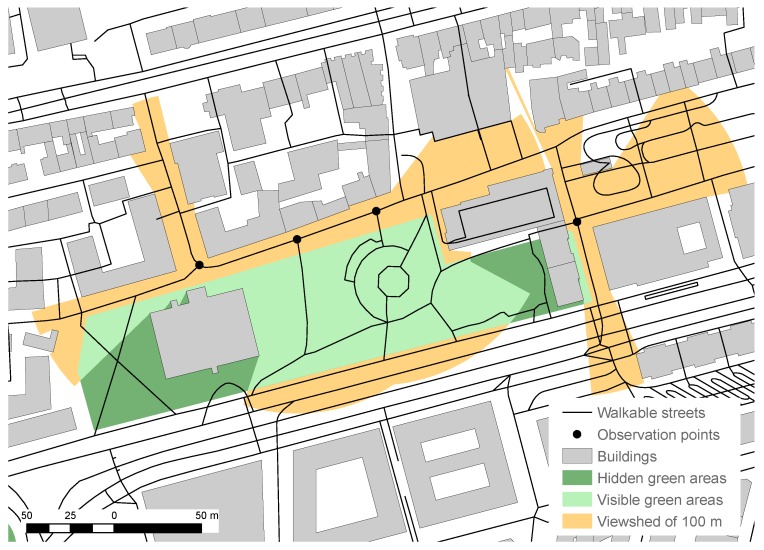
Example of the visible green areas inside the viewsheds of the 100 m radius from the four observation points.

The computation of the greenness of street segments was preceded by the selection of OSM features with at least one of the tags presented in Table 2 that are considered to indicate green areas. Observation points were then established at the beginning and end of each street segment shorter than 50 m. For longer street segments, *n* = lengthofthestreetsegment(m)/50 equidistant observation points was established (in practice, *n* was rounded to its closest integer). Subsequently, the viewshed in a radius of 100 m was computed for each of these observation points. These viewsheds were merged and the green areas intersecting the merged viewshed were extracted. This process is depicted in Figure 3. Finally, the relative visible green area inside the merged viewshed was computed. The greenness factor *g* of each street segment *i* was then given by

(1) $$g_{i}=\frac{\mathrm{Length}\;\mathrm{of}\;\mathrm{street}\;\mathrm{segment}\;i}{\mathrm{Relative}\;\mathrm{area}\;\mathrm{of}\;\mathrm{green}\;\mathrm{areas}\;\mathrm{inside}\;\mathrm{the}\;\mathrm{merged}\;\mathrm{viewshed}+0.5}.$$

The reason for adding 0.5 into the denominator of Equation (1) was that, without it, street segments with little visible green areas around them would get very high $g_{i}$ factor values. This is because dividing any number by a value close to zero leads to the manifold multiplication of that number. The effect would be the extraction of absurdly longer routes that do not make any sense in terms of the number and direction of turns. The addition of 0.5 in the denominator thus sets the constraint that $g_{i}$ will be, at maximum, twice the length of the street segment *i*. This value was defined empirically during exploratory tests.

**Figure 4:**
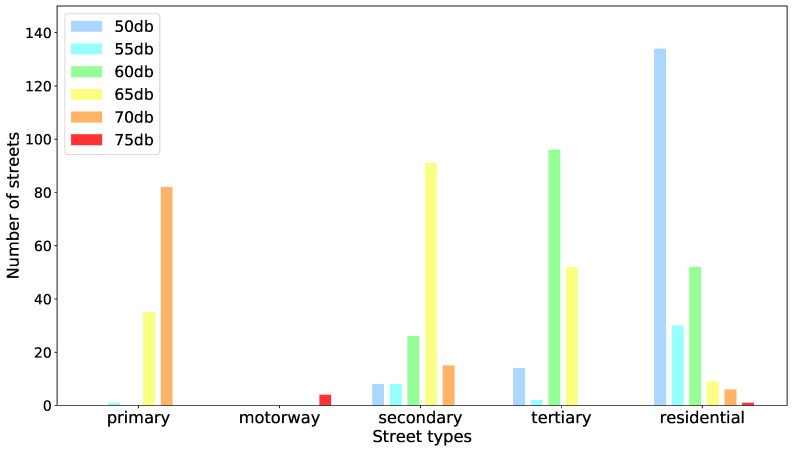
Distribution of noise intensity levels for each OpenStreetMap highway type. The noise intensity data from each street segment was collected and provided by the city of Heidelberg (Germany).

Regarding the quietness factor, the street segments in OSM were grouped into two categories according to their highway/street type tag, namely, ‘noisy’ and ‘less noisy’. As shown in Table 3, the OSM highway type tags were associated with noise factors of 1 or 2. Following, the quietness factor of each street segment was computed by dividing its length by the noise factor associated with its highway type tag. Although this is a very simplified way to describe the quietness of streets, we took into account that the main source of noise in urban areas is car, truck, and bus traffic [34]. Furthermore, based on official noise intensity data from the city of Heidelberg (Germany)—the test-site of our system’s prototype—we observed a consistent correlation of higher and lower noise intensity levels with street segments associated with noise factors 1 and 2, respectively. Figure 4 depicts the distribution of noise intensity levels for each highway type from OSM. Based on a visual analysis of these distributions, noise factors were associated with OSM highway types according to Table 3.

The cost function based on which the system extracts the pedestrian routes considers the length as well as the sociability, greenness, and quietness factors of the street segments. As explained in Section 3, each term is normalized and then multiplied by a user-defined weight which is discretized into eleven levels from 0 to 10. The four normalized and weighted factors are then summed. Thus, the final weight $W_{i}$ of a street segment *i* is given by

(2) $$W_{i}=w_{l}\hat{l_{i}}+w_{s}\hat{s_{i}}+w_{g}\hat{g_{i}}+w_{q}\hat{q_{i}},$$

where $\hat{l_{i}}$, $\hat{s_{i}}$, $\hat{g_{i}}$, and $\hat{q_{i}}$ are, respectively, the normalized length, greenness, sociability, and quietness of a street segment *i*. The terms $w_{l}$, $w_{s}$, $w_{g}$, and $w_{q}$ are the respective user-defined factor weights. As mentioned, these weights are set by means of slide-bars available in the system’s interface.

The lengths of the street segments were considered in the computation of the greenness, sociability, and quietness factors, because our intention was that the alternative routes extracted by the system would not be significantly longer than the shortest routes but have significantly less noisy meters as well as more green areas and social places, depending on the user-defined factor weights and the city structure, of course. Thus, we assumed that, most of the time, users of our system will not be willing to make very long detours for the sake of pleasant walking, which is usually the case in more rare situations, like touristing or city exploration. Our assumption is corroborated by the seminal work of Seneviratne et al. [6] where evidence is given that distance is the strongest factor in pedestrian route choice. A more practical justification is that, even if the length factor has a strong weight, very long and complex routes are obtained if the length is not also considered in the computation of the other factors.

## 4. Evaluation of the System’s User Perception

The proposed system for generating customized pleasant pedestrian routes based on OSM data was also evaluated with respect to its usefulness, usability, and controlability/transparency, which are aspects proposed by Pu et al. [47] for evaluating recommendation systems from the perspective of the users. The usefulness aspect refers to the users’ perceptions on the utility of the system to their everyday lives. The usability aspect refers to the perceived easiness of use of the system, i.e., to how intuitive and easy it is for users to interact with the system. Controlability refers to the extent to which the system enables users to express their preferences and needs and thus control the production of customized results delivered by the system. Transparent recommendation systems enable users to understand the relation between the user-defined settings and the outcomes recommended by the system. They also provide the necessary information to assist users in their decision to accept the system’s recommendation or to further adjust their settings/preferences.

In order to evaluate these aspects of the system, a user-survey was designed. The survey was divided in four parts. In the first part, questions related to the *potential* utility of the system were asked. That means, without knowing what the system is about, participants were asked the following main questions:

- **Q1**: How many times a month do you usually use a navigation system for walking?
- **Q2**: When walking to a destination, how often do you choose a route that you like best, even though it might not be the shortest one?
- **Q3**: Would a pedestrian navigation system that suggests longer but more pleasant and interesting routes be useful to you?
- **Q4**: When walking to a destination, how frequently do you prefer streets with (a) green areas, (b) social places, and (c) less traffic and less noise pollution?

In the second part of the survey, the system is presented. Based on the usability inspection method of cognitive walk-through [48], we presented the participants of the survey, the chronological steps undertaken by the user, and the respective responses of the system. In this part of the survey, participants were presented with the four system components shown in Figure 1. Thus, participants were given a clear idea of how the system would be used as well as on the purpose of its components.

In the third part of the survey, questions related directly to the system itself were asked, as follows:

- **Q5**: In your opinion, is the routing system presented to you useful?
- **Q6**: Does the routing system seem to be easy to use?
- **Q7**: Is expressing your route preferences to the system easy and intuitive?
- **Q8**: Is the provided information comparing the characteristics of the shortest and alternative routes useful to you?
- **Q9**: Does the provided information comparing the characteristics of the shortest and alternative routes help you decide which route to take?
- **Q10**: Does the system give you control in discovering routes that match your preferences regarding green areas, social places, and quiet streets?

In the forth and last part of the survey, we asked the participants’ opinions related to the limitations and possible improvements of the system. This part of the survey was very important for identifying users requirements with respect to providing a pedestrian routing system with customized pleasant routes destinations.

## 5. Experiment Design

To evaluate the routing cost function presented in Section 3.2, we randomly generated one-thousand trips, i.e., origin–destination pairs, with the shortest walking distances being between 1000 and 4000 m. We defined this distance interval based on the work of Yang and Diez-Roux [9], in which it is reported that many people walk for more than one mile (≈1800 m) in recreational situations. These one-thousand trips were all within the city of Heidelberg (Germany), which is one of the most touristic cities in Germany.

For each trip, besides the shortest walking route, fifteen alternative routes were extracted considering the factor weight sets presented in Table 4. Given the impossibility of analyzing all 14,640 possible factor weight combinations, we chose these fifteen, believing they are the most representative of (or at least similar to) weight sets commonly defined by users. The first twelve weight sets from Table 4 were chosen as they have the main proportions between the weights of the length and one of the other three factors (i.e., weight set IDs 1–4 for length and greenness, 5–8 for length and sociability, and 9–12 for length and quietness). Factor weight sets 13 and 14 were tested as they are the most generic combinations of the weights (namely, of equal weights) of the length and two of the other three factors. Finally, factor weight set 15 is the combination of all four factors with equal weights.

The total distance, greenness, sociability, and quietness factors of the shortest route of each of the one-thousand trips were computed. The total distance and factors with non-zero weights were also computed for each of the fifteen alternative routes from each trip. Then, the shortest route was compared with the alternative routes from the respective trip using the following metric:

(3) $$p_{i}=\left(\frac{F_{i}^{a}}{F_{i}^{s}}-1\right)*100,$$

where $F_{i}$ represents the sum of one of the *i* factors, i.e., length, greenness, sociability, and quietness, over all street segments of the entire route. $F_{i}^{s}$ and $F_{i}^{a}$ are the values of the summation for the shortest and one of the alternative routes, respectively. In the case of Equation (3), the greenness factor is not the same as that computed with Equation (1). Rather, it represents the lengths of the routes’ street segments multiplied by the relative amount of green area inside their viewshed (see Section 3.2). The sociability factor refers here just to the total number of social places along the route. The quietness factor refers to the total length of the route along streets considered to be noisy. Compared with Equation (3), the quietness of the shortest and alternative routes of each trip, the fraction was inverted so that the metric describes the percentage of meters along noisy streets that the alternative route has.

Regarding the evaluation of the system’s user perception, the user-survey presented in Section 4 was taken by 77 females and 79 males. The survey request was sent to all students and employees of the Geography Institute of the University of Heidelberg.

## 6. Results

### 6.1. On the Intrinsic Evaluation of the Routing Cost Function

As mentioned in Section 5, for each of the one-thousand randomly generated trips, the shortest route as well as fifteen different alternative routes were generated by applying the factor weight sets presented in Table 4. For each trip and factor weight set, the $p_{i}$ metric (Equation (3)) was computed for the length as well as for the factors with non-zero weights of each set.

Table 5 presents the descriptive statistics of the $p_{i}$ metric from the non-zero weighted factors of all fifteen factor weight sets. These statistics were computed from the one-thousand randomly generated trips. It can be noticed that, on average, the alternative routes were less than 10% longer than the shortest route of their respective trips. In fact, with the exception of those generated with weight sets 4, 8, and 12, the alternative routes were, on average, less than 4% longer than the shortest routes of all the one-thousand trips. The standard deviation and the 80th percentile statistics of the $p_{i}$ metric for the factor length also indicated that the alternative routes generated with all fifteen weight sets were, in general, only slightly longer than their respective shortest routes.

Besides most of the alternative routes being only slightly longer, they are, in general, much more social, green, and quiet than the shortest route of their respective trips. The factor weight set 3, for example, generated eight-hundred of the one-thousand trips alternative routes that were less than 7% longer but more than twice as green as the respective shortest routes. Regarding the sociability factor, most alternative routes generated with factor weight set 8 were less than 10% longer but had up to 350% more social places than their respective shortest routes. Regarding the quietness factor, similar numbers for the extra length and fewer noisy meters were obtained. Factor weight set 11, for example, generated alternative routes that were generally less than 6% longer than the respective shortest routes. However, these routes had about 160% less meters along streets considered to be noisy (see Table 3).

Factor weight sets from 1 to 12 have non-zero weights applied to only one of the sociability, greenness, or quietness factors. Based on Table 5, it can be argued that these weight sets produced slightly longer but much more social, greener, and quieter alternative routes, as required. Factor weights 13, 14, and 15, on the other hand, have non-zero weights applied to more than one of the sociability, greenness, and quietness factors. These weight sets also produced, on average, only slightly longer routes (i.e., less than 2%) that were however significantly more social and greener as well as quieter than the respective shortest routes. This can also be observed based on the 80th percentile statistic of the $p_{i}$ metric computed for these factors and weight sets based on the one-thousand randomly generated trips.

**Figure 5:**
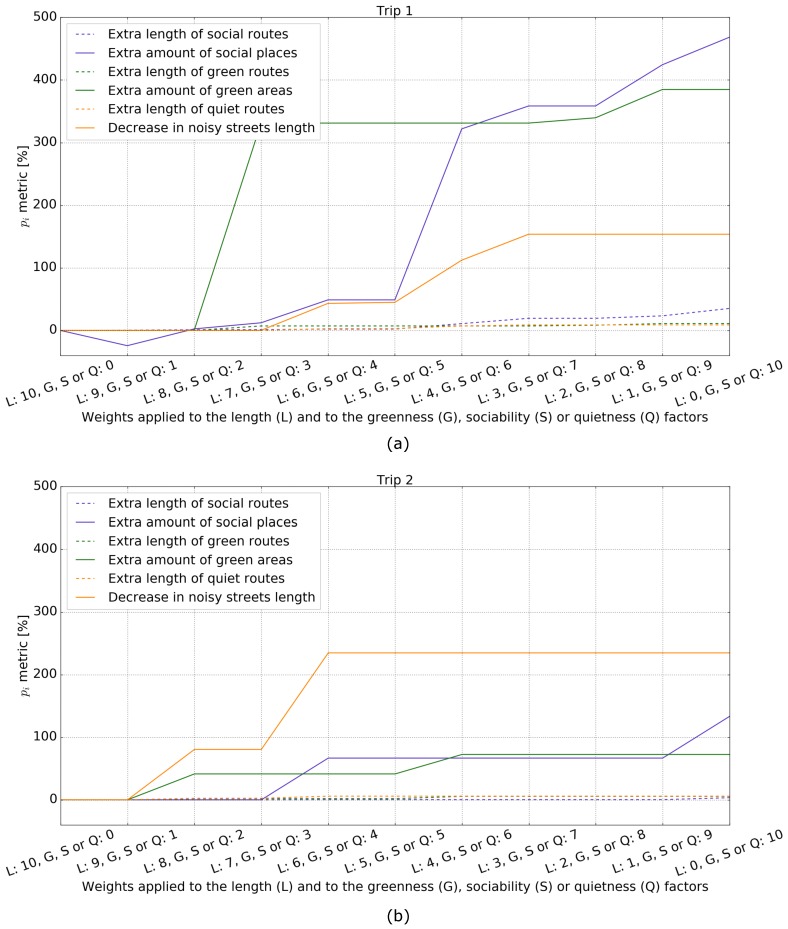
Graph plots of the $p_{i}$ metric computed for two trips, shown in (**a**) and (**b**), and the eleven discrete weight pairs summing to 10, i.e., (10,0),…,(0,10). Each pair of weights were applied to the length (L) and to the greenness (G), sociability (S), or quietness (Q) factor separately.

Figure 5 shows the $p_{i}$ metric of the length as well as the sociability, greenness, and quietness factors computed from alternative routes generated with all eleven combinations of discrete weights summing 10, i.e., (0,10), (1,9), …, (9,1), (10,0) for two specific trips. These pairs of weights were applied to the length and to one of the other three factors. It can be seen that the extra length of the alternative routes generated with all eleven weight pairs was always only slightly longer than the shortest route, whereas the $p_{i}$ of the other factors increased significantly as their weights increased from 0 to 10. Therefore, Table 5 and Figure 5 together demonstrate that the routing cost function implemented in our system is expected to generate acceptably longer and significantly more social, greener, and quieter alternative routes, depending on what the factor weight is.

**Figure 6:**
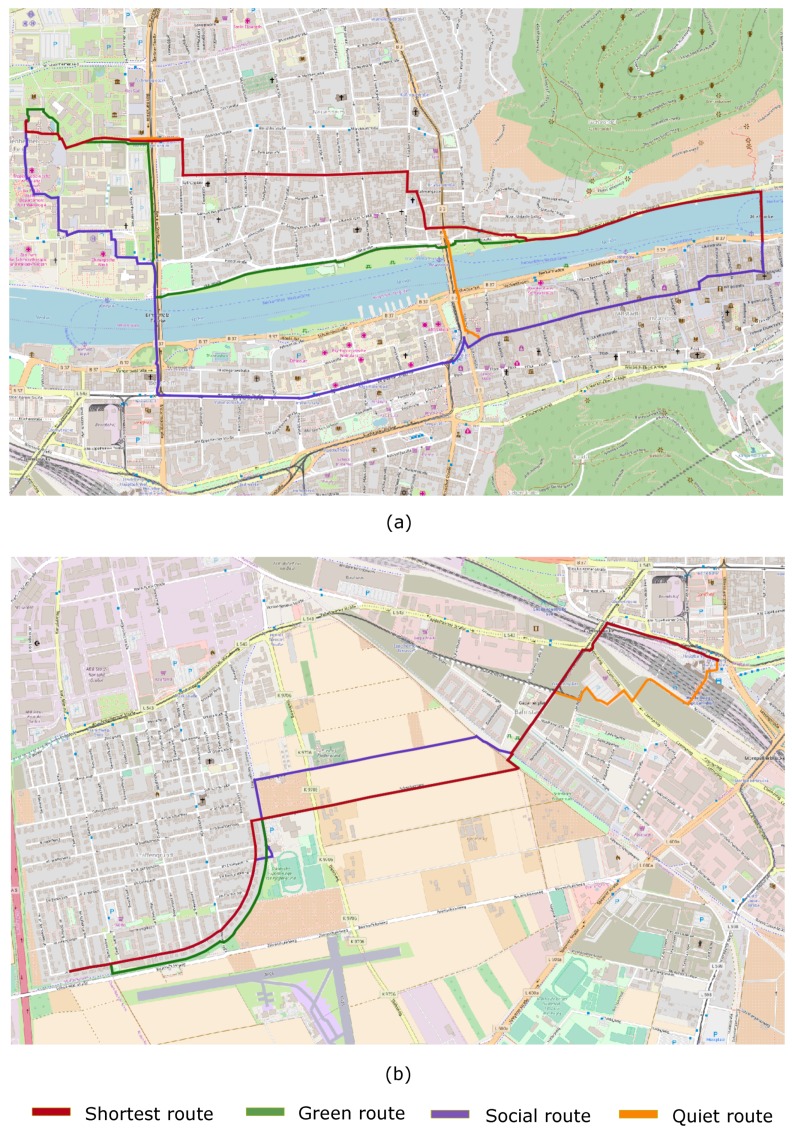
Shortest and alternative walking routes generated for trips 1 and 2 (Figure 5), shown in (**a**) and (**b**) in Heidelberg (Germany). The alternative routes were generated by setting a weight of 2 to the length factor and 8 to the other three factors separately.

Figure 6 depicts the shortest as well as alternative routes generated for the two trips from Figure 5 by applying a weight of 2 to the length factor and of 8 to the other three factors separately. It can be seen that the alternative routes of Figure 6a were similar in length but very different in terms of the actual routes. The trip from Figure 6b goes from a residential area to the Heidelberg Central Station. The shortest route crosses some agricultural fields. There are no significantly more interesting routes that do not deviate too much from the shortest route. In this case, the system, desirably so, suggests slightly different routes instead of detours that few or no person would prefer.

### 6.2. On the System’s User Perception

Based on the survey taken part by 156 participants, the system’s usefulness, usability, and controllability/transparency were evaluated. Table 6 presents the results of the ten questions from the survey presented in Section 4.

Regarding the aspect of usefulness, it can be seen from Table 6a that 55% of the participants claimed to use a routing system for walking at least twice a month, while only 9% said they do not use such a system at all. Table 6b shows that more than 50% of the participants stated that they always or frequently choose walking routes they prefer, even though these routes might not be the shortest ones. There might be many different factors influencing this choice with strengths varying according to an individual’s preference, time of the day, and other circumstances. More than 85% of the participants, however, claimed that their route choice is always or frequently influenced by the presence of green areas and the avoidance of noisy streets with intense traffic. The participants seemed to be less frequently influenced by the presence of social places. However, less than 22% of them claimed to be influenced only rarely or never by this factor (Table 6c). Question 3 (Q3) also reiterates the usefulness of a system that suggests pleasant pedestrian routes (Table 6d). Almost 70% of the participants shared the opinion that such a system would be useful for them. Interestingly, after the concept of our system is presented to them, the percentage of participants that agreed or strongly agreed on the usefulness of the system increased to more than 85%, as the answers to question 5 (Q5) on Table 6d indicate.

Concerning the usability of our proposed system, as shown by the results from Question 6 on Table 6d, more than 90% of the participants agreed or strongly agreed that it would be easy to use it. Also, more than 85% of the participants agreed or strongly agreed that expressing their route preferences with respect to the four factors the system considers is easy and intuitive. This is shown by the results from Question 7 on Table 6d.

Questions 8 and 9 from the survey were asked with the intention of inspecting the users’ perceptions on the transparency of the system. They refer specifically to the information shown on the system’s interface about the total length, greenness, number of social places, and total length of the noisy streets that the shortest and the alternative route have. More than 80% of the participants agreed or strongly agreed that this information is useful and assists their decisions on whether to take the shortest or an alternative route (Table 6d).

Regarding Question 10, more than 85% of the participants agreed or strongly agreed that the system would give them control in discovering routes that reflect their preferences with respect to the four factors considered by the system (Table 6d). This control is exerted through slide-bars with which the weights the factors receive in the routing cost function are defined.

Participants on the survey were also asked about the extent to which certain conditions influence their decision to take a more interesting and pleasant route. About 74% of the participants said time availability influences their decision to a great extent. Almost 90% of them claimed that the extra length of the alternative route influences their decision somewhat or to a great extent. More than 80% of the participants said their decision is at least somewhat influenced by their mood and by how they feel at the time. Most interesting though is that more than 90% of them claimed that their decision on whether to take the alternative route depends somewhat (39%) or to a great extent (52%) on how much more interesting and pleasant it is. This supports the importance of informing users on the length as well as the greenness, quietness, and number of social places of the shortest and alternative routes. It also justifies the necessity of the system to let users interactively define the weight of each factor according to their circumstances.

Regarding future improvements to the system, among the expressed opinions, almost 50% of the participants suggested including the possibility of generating safe routes. Thirty-eight percent of the participants agreed that letting users actively define what types of social places they are interested in would be a relevant improvement as well. The same percentage of participants suggested that the system should only consider social places that are certain to be open at the time of the route request. In the feedback given by the participants in free-text form, they consistently mentioned the wish to visualize the locations of most attractive social places and green areas and which are the most quiet streets. The participants also reiterated their suggestion that the system should recommend safe routes.

As the main limitations of the system, among the expressed options, 44% of the participants thought the system did not consider other factors that influence their route choice. However, only 11% did not feel convinced that the alternative route would satisfy them with respect to green areas, social places, and quieter streets.

## 7. Summary and Discussion

We presented a system for generating pleasant pedestrian routes based on OSM data. A route is considered pleasant when having green areas and social places as well as streets with less traffic noise. An important feature of the system is that it allows users to interactively define the weights of these factors in the extraction of the customized route. The system also informs users about the length, greenness, number of social places, and total length of noisy streets of the shortest and customized routes. This enables them to make a more aware decision on whether to take one of the two routes or to further edit the factor weights. In our opinion, such an user-system interaction is important to the extent that pedestrian route choice is subjective and dependent on the circumstances at the moment of the route request. The range of user types and situations in which the system can be utilized is therefore broadened by the possibility of generating customized routes in an interactive way.

One of the limitations of the system is that so far the routing cost function does not internally consider the time of the day that the route request is made. This makes the system enable to disregard social places that are closed at the time. However, this technical limitation can easily be overcome as long as the venues opening hour information is available in OSM. Besides, users are always aware of the time of the day and can define the factor weights in a way that leads to the desired route characteristics. For example, by avoiding green areas and preferring more social and less quiet routes during the night, safer routes can potentially be obtained.

The fact that the system is based entirely on data from OSM is, at the same time, a shortcoming and an advantage. It is a shortcoming because OSM data might be incorrect, missing, or incompletely available in some areas. It is, on the other hand, an advantage because the system can potentially be implemented for any city of the world that is already mapped in OSM. Because OSM is becoming more and more established as a reliable source of geo-data for different purposes, GIS-applications based on OSM are expected to be sustainably maintained and operational long-term. Furthermore, the potential to improve such applications lies not only on the increasing completeness and reliability of the data but also in the fact that, since OSM has a well-established data structure, researchers from different fields and geographic regions will have less difficulty improving and extending these applications in a collective and cooperative way.

To the best of our knowledge, our work is the first to quantify important pedestrian route choice factors based on OSM data and integrate them into a routing cost function that, according to our intrinsic analysis, generates routes that are slightly longer but significantly more pleasant. We also presented the prototype of the system’s interface that is already integrated into OpenRouteService, a widely used web-service for other navigation purposes.

Lastly, the results from the applied user-survey allow us to conclude that the system is useful, transparent, and intuitive to use. The survey also indicated what other functionalities users would like the system to have, thus giving us clear directions for upcoming developments in the system.

## Figures and Tables

**Table 1:** OpenStreetMap tags considered to indicate a third place.

Key	Values
amenity	cafe, bar, pub, restaurant
shop	bakery, convenience, supermarket, mall, department_store, clothes, fashion, shoes
leisure	fitness_centre

**Table 2:** OpenStreetMap tags considered to indicate green areas.

Key	Values
amenity	grave_yard
landuse	allotments, cemetery, farmland, forest, grass, greenfield, meadow, orchard, recreation_ground, village_green, vineyard
leisure	garden, golf_course, nature_reserve, park, pitch
natural	wood, scrub, health, grassland, wetland
tourism	camp_site

**Table 3:** Assignment of highway type tags from OpenStreetMap to noise factors.

OSM Tag (Key = Value)	Assigned Noise Factor
highway = motorway	1
highway = trunk	1
highway = primary	1
highway = secondary	1
highway = tertiary	2
highway = residential	2
pedestrian = yes	2

**Table 4:** Sets of factor weights applied to generate alternative routes for the one-thousand randomly generated trips.

Weights Set ID	Length	Greenness	Sociability	Quietness
1	7	3	-	-
2	5	5	-	-
3	3	7	-	-
4	-	1	-	-
5	7	-	3	-
6	5	-	5	-
7	3	-	7	-
8	-	-	1	-
9	7	-	-	3
10	5	-	-	5
11	3	-	-	7
12	-	-	-	1
13	1	1	1	-
14	1	1	-	1
15	1	1	1	1

**Table 5:** Descriptive statistics of the $p_{i}$ metric (Equation (3)) computed for the length as well as the non-zero weighted factors of the fifteen weight sets presented in Table 4. These statistics were computed over the one-thousand randomly generated trips. * L, G, S, and N stand for the length, greenness, sociability, and noisiness factors, respectively.

Weight Set ID	Factor *	Mean	SD	Max.	80th Perc.
1	L	0.51	1,25	9.83	0.62
1	G	85.7	676.6	17,155	36.1
2	L	1.51	3.11	27.9	2.1
2	G	175.0	1007	17,155	80.4
3	L	3.51	5.85	37.3	6.36
3	G	373.3	2800	75,203	164.3
4	L	9.6	13.7	89.8	15.5
4	G	997.1	8110	211,610	351.1
5	L	0.53	1.25	15.6	0.76
5	S	83.9	347	5400	69.8
6	L	1.48	2.88	23.8	2.38
6	S	157.6	703	16,900	125.2
7	L	2.69	4.49	33	4.30
7	S	221.1	916	17,800	200
8	L	5.54	8.47	56.5	9.53
8	S	349.5	1194	19,500	350
9	L	0.76	1.42	12.6	1.36
9	N	512.8	7226	176,596	68.9
10	L	1.69	2.82	20.1	3.09
10	N	639.6	7690	176,596	117.1
11	L	2.91	4.13	31.2	5.82
11	N	662.5	7690	176,596	158.6
12	L	4.86	6.27	45.5	9.1
12	N	706.2	7705	176,596	189.9
13	L	0.91	1.60	15.6	1.52
13	G	93.6	879.8	17,155	25.1
13	S	67.0	288.1	5400	60.0
14	L	1.96	3.23	33.2	3.20
14	G	176.5	1103	17,155	82.1
14	N	645.8	7708	176,596	90.2
15	L	1.37	2.22	16.6	2.47
15	G	126.8	936.8	17,155	41.0
15	S	71.0	301.6	5800	75.0
15	N	616.4	7691.8	176,596	64.5

**Table:** (**a**)

Q1	
Four times or more	16.0%
About two or three times	39.7%
Once	14.1%
Less than once	21.2%
I do not use a routing system for walking	9.0%

**Table:** (**b**)

Q2	
Always	7.1%
Frequently	43.5%
Occasionally	33.1%
Rarely	14.9%
Never	1.3%

**Table:** (**c**)

Q4			
	**Green Areas**	**Social Places**	**Less Noise and Traffic**
Always	44.44%	16.45%	56.49%
Frequently	41.83%	30.92%	29.87%
Occasionally	11.11%	30.92%	9.74%
Rarely	1.96%	15.79%	2.60%
Never	0.65%	5.92%	1.30%

**Table:** (**d**)

	Q3	Q5	Q6	Q7	Q8	Q9	Q10
Strongly agree	19.4%	30.9%	34.2%	30.9%	30.2%	23.0%	33.8%
Agree	48.4%	55.0%	58.4%	54.4%	52.3%	58.1%	51.4%
Neither agr. nor disagr.	24.5%	11.4%	5.4%	12.8%	14.1%	14.9%	11.5%
Disagree	7.1%	1.3%	2.0%	2.0%	2.7%	4.1%	2.7%
Strongly disagree	0.6%	1.3%	0.0%	0.0%	0.7%	0.0%	0.7%
